# Impact of an educational flyer and sensitization on performance-enhancement attitudes of bodybuilders in United Arab Emirates

**DOI:** 10.12688/f1000research.130700.1

**Published:** 2023-02-23

**Authors:** Dixon Thomas, Adhnan Abdul Shabeek, Hala Ahmed, Malak Mohammed, Marina Kawaguchi-Suzuki, Ashley Anderson, Aji Gopakumar, Reema Alhosani, Sherief Khalifa, David Mottram

**Affiliations:** 1 College of Pharmacy, Gulf Medical University, Ajman, United Arab Emirates; 2 School of Pharmacy, Pacific University, Oregon, USA; 3 International Sports Pharmacists Network, Colorado, USA; 4 Department of Research, Emirates Health Services, Dubai, United Arab Emirates; 5 National Antidoping Agency, Dubai, United Arab Emirates; 6 Pharmacy and pharmaceutical science, Liverpool John Moores University, Liverpool, UK

**Keywords:** Bodybuilders, performance enhancement, doping, educational intervention

## Abstract

**Background:** A high proportion of bodybuilders use supplements to improve performance, with some turning to prohibited substances and methods. The attitudes of bodybuilders towards performance enhancement may be gauged through surveys such as the Performance Enhancement Attitude Scales (PEAS). Educational interventions are recommended as part of anti-doping measures. The objective of this project was to assess the impact of a pharmacy-led intervention using an antidoping educational flyer and the performance enhancement attitude scale to measure the attitude of bodybuilders in the United Arab Emirates (UAE).

**Methods:** The PEAS eight-item short form questionnaire was administered to male bodybuilders in the UAE. The PEAS was conducted before and after administration of an educational flyer concerning the problems associated with supplement use among bodybuilders. The Wilcoxon Signed-Rank and Kruskal Wallis tests were used for data analysis.

**Results:** A total of 218 bodybuilders, who reported taking dietary supplements, filled out the survey both pre and post viewing the antidoping educational flyer. A difference was observed between the full-time professional bodybuilders, students, and part-time bodybuilders with other primary occupations (p-value <0.05). In addition, PEAS score decreased among the study population for all eight PEAS items (p-value <0.05).

**Conclusions:** The pharmacy-led intervention using an antidoping educational flyer and sensitization by PEAS achieved more favorable scores, suggesting a significant shift of opinion toward avoiding use of performance enhancing substances among the bodybuilder study population. More research is required on sustaining the attitude and demonstrating the impact on doping behavior.

## Introduction

Exercise in gymnasiums is a feasible way to be physically active in the United Arab Emirates (UAE). Chronic diseases pose significant public health issues among the UAE population. ^1^ In particular, attitudes about obesity management need improvement. ^2^ There is a lack of public awareness of the importance of engaging in physical activity in the UAE, resulting in high levels of sedentary behavior among young adults. ^3^ Promotion of the benefits of physical activity are therefore required among the UAE population. ^4^

It is acknowledged, however, that gymnasium use may progress from simple exercise regimens to a desire for image enhancement that leads some to bodybuilding. ^5^ Among gymnasium users in the UAE, bodybuilding, either for non-competitive (recreational) or competitive purposes, is a popular sport. The motivation for bodybuilding is manifold and ranges from the improvement of body image and well-being through to participation in competitive sport. ^6^

Many gymnasium-users take supplements to improve their performance or image enhancement. In a study in Sharjah, it was shown that about half of the men exercising in gyms were using dietary supplements. ^7^ In another study, in Dubai, it was reported that people who were consuming dietary supplements had a high level of knowledge about the supplements that they used and consequently, adverse events were infrequent. ^8^

Dietary supplements are used widely among bodybuilders. However, in addition, some bodybuilders may use hormonal products, which pose a potential health risk ^9^ and may lead to illegal doping in the competitive areas of bodybuilding. ^10^^,^^11^ Some bodybuilders in the Gulf region reportedly use anabolic steroids, ^12^ reflecting an increased prevalence of anabolic-androgenic steroid (AAS) use generally across the Eastern Mediterranean Region. ^13^

Many factors contribute to performance enhancement by bodybuilders and its progressive normalization. ^14^ There is a growing body of evidence which suggests that anabolic androgenic steroids are used globally by a diverse population, with varying motivations, including bodybuilders. ^15^ As the trend of bodybuilding is transforming to create a well-defined and moderately muscular body, it is speculated that fitness doping is becoming increasingly common. ^16^ However, the boundary between natural performance enhancement and doping might be blurred for the bodybuilders. Considering the potential adverse effects of AAS use on health, it is important to consider intervention strategies to prevent misuse, both in sport and in the general population. ^17^ Such strategies include targeted education and a greater understanding on doping attitudes through tools such as the Performance Enhancement Attitude Scale (PEAS). ^18^

Pharmacists are well-placed and highly accessible resources of drug information for the public. The opportunity exists to increase pharmacy-led initiatives that support the antidoping movement.

Antidoping assessments and education raise awareness of doping. It is suspected that bodybuilders might be sensitized to the problems of performance enhancement attitude just by filling out the pre-intervention survey. This awareness of perceptions associated with doping, in addition to the educational flyer developed for antidoping education for bodybuilders, was expected to influence attitudes toward higher concern for the risk of performance enhancement substances among bodybuilders in the study population.

### Aim

The aim of this project was to assess the impact on local bodybuilders’ views of using substances for performance enhancement before and after a pharmacy led intervention using an antidoping educational flyer, as measured by the PEAS.

## Methods

### Research design

**Figure 1.**
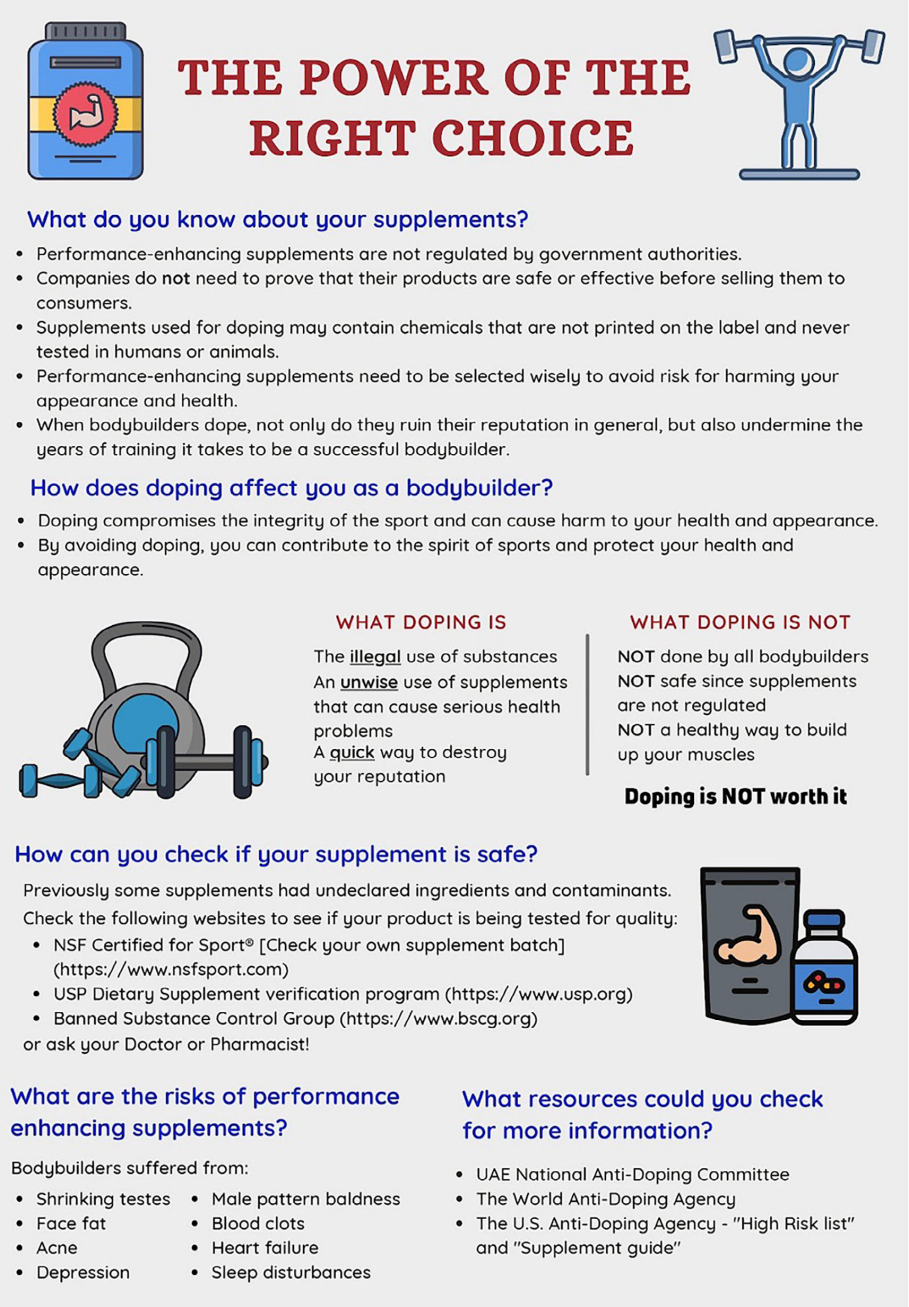
Educational flyer.

The study used a pre-post intervention design. Attitudinal data were collected using a PEAS. An antidoping educational flyer was designed by a sports pharmacy team with a simple infographic design and clear messaging on the risk of doping. Sports pharmacy team members then administered the intervention by presenting the educational flyer to bodybuilders enrolled in the study. The educational flyer is included as Figure 1. The educational flyer was provided to bodybuilders prior to the post-intervention survey. The second PEAS survey was administered approximately one month after the first administration of the PEAS survey among the study population.

### Study population

The study population included self-described bodybuilders in the UAE. The study did not use any brand name or manufacturer names of any performance enhancement substance. Being a male bodybuilder who uses nutritional supplements and consenting to participate in the study were the inclusion criteria. Any level of bodybuilders, including recreational, amateur, or professional athletes, were recruited. Bodybuilders who were not regularly practicing in their gym, could not read English, or were not living in the UAE were excluded from the study population. Background data was collected from the bodybuilders on their level of involvement in bodybuilding and nutritional supplements used.

### Sample size, survey technique, and settings

The sample size was calculated using a formula that considers the population size, margin of error, and sampling confidence level. Survey Monkey's sample size calculator accounts for the three factors mentioned above. The bodybuilder population in UAE is approximately 500,000, based on data from Dubai World Trade Centre. Using a 95% confidence interval and margin of error of eight, the calculated sample size was 151 bodybuilders. The sampling strategy included distributing a pre-intervention survey to registered study participants through email and WhatsApp. A post-intervention survey following the educational intervention was sent only to those who completed the pre-intervention survey. Each survey was conducted using Google Forms and paper-based by member of the sports pharmacy team visiting local gyms. The total duration of the study was one year.

### Study instrument & validation procedure

A modified version of the PEAS, known as the eight-item short form, was used as the survey instrument. ^18^^,^^19^ For each item a six-point Likert scale of strongly disagree (1), disagree (2), slightly disagree (3), slightly agree (4), agree (5), and strongly agree (6) was used. The PEAS is an internationally validated instrument. Only in required items, the word “bodybuilding” was added to connect to the athletes of this study population. The meaning of doping was added in parenthesis when it appeared for the first time on the survey. In addition to some background enquiries, the pre- and post-survey questionnaire included the following eight-items.

1. Legalizing performance enhancements would be beneficial for sports or bodybuilding (competitive or non-competitive).
2. Doping (consuming prohibited substances) is necessary to be competitive.
3. The risks related to doping (consuming prohibited substances) are exaggerated.
4. Bodybuilders should not feel guilty about breaking the rules and taking performance-enhancing drugs.
5. Doping is an unavoidable part of competitive sport/bodybuilding.
6. Doping is not cheating since everyone does it.
7. Only the quality of performance should matter, not the way bodybuilders achieve it.
8. There is no difference between drugs and dietary supplements that are all used to enhance performance.

A higher number for the final score of the PEAS suggests agreement with the statements that support use of performance enhancing substances or the doping culture.

### Ethical consideration

Ethical approval to conduct the research was granted by the Institutional Review Board of the Gulf Medical University (IRB/COP/STD/73/Oct-2021). Written informed consent was obtained for participating in the study. No personal identifiers of study participants were collected except email and phone numbers for sending the post-intervention survey. Such contact details were kept confidential. No brand names of bodybuilding supplements or medications were mentioned in the study. All confidential information was maintained by the authors from Gulf Medical University and not shared with the authors from other organizations in this study.

### Data analysis

Descriptive and analytical statistical tools were used to compare the attitudes of bodybuilders before and after the pharmacy-led educational intervention. Wilcoxon Signed-Rank test was used to assess performance enhancement attitude (PEA) scores before and after the intervention. Kruskal Wallis tests were used to find the statistical significance of the pre-post intervention among different groups. SPSS version 26 (Armonk) was used for all the analyses.

## Results

Of the 322 bodybuilders who filled out the pre-intervention survey, 254 completed the post-intervention surveys (79% response rate). ^29^ For the analysis, 22 respondents were removed due to the lack of matching email or mobile numbers used as identifiers in the pre-survey to post-survey tracking. Other respondents were excluded from the study by not being located in the UAE or reporting they were not consuming dietary supplements. A final total of 218 respondents were used for the data analysis. The majority (98.2%) of study participants found the antidoping educational flyer to be informative, while four (1.8%) participants responded that it was not informative enough.

The study population constituted of 80 bodybuilders of non-sports employment, 16 full-time bodybuilders, 111 university students, and 11 school students. All were male. The Kruskal Wallis test showed a significant difference in the post-intervention (p-value 0.002) but not in pre-intervention (p-value 0.07) among the four subgroups of this study population.

When the average score is compared among the four groups by the median test, the median (average) of the overall pre-score varied across the groups (p-value <0.001), but the average of the overall pre-score is almost the same between the groups (p-value 0.07). It implies that the post score changed significantly when compared with the average pre-score, but the average attitude level became stable and is matched among different types of participants.

With the intervention of the educational flyer and sensitization, the PEA was changed favorably towards recognition of concerns associated with doping among the participants. Agreement to pro-doping statements in the PEAS survey decreased, meaning disagreement increased with the intervention as shown in Table 1. For tabulation, “strongly agree” and “agree” were combined; likewise, responses of “slightly agree” and “slightly disagree,” and “disagree” and “strongly disagree” were combined.

**Table 1.** Percentage responses before and after intervention.

Modified (for bodybuilding) Performance Enhancement Attitude Scale (PEAS) 8-item short form	Timing of survey to intervention	Strongly Agree & Agree		Slightly Agree & Slightly Disagree		Disagree & Strongly Disagree	
		N	%	N	%	N	%
1. Legalizing performance enhancements would be beneficial.	Before	89	40.8	59	27.1	70	32.1
	After	20	9.2	39	17.9	159	72.9
2. Doping is necessary to be competitive.	Before	58	26.6	52	23.9	108	49.5
	After	15	6.9	40	18.3	163	74.8
3. The risks related to doping are exaggerated.	Before	52	23.9	75	34.4	91	41.7
	After	12	5.5	52	23.9	154	70.6
4. Bodybuilders should not feel guilty about breaking the rules and taking performance-enhancing drugs.	Before	69	31.7	44	20.2	105	48.2
	After	18	8.3	44	20.2	156	71.6
5. Doping is an unavoidable part of the competitive sport/bodybuilding.	Before	76	34.9	53	24.3	89	40.8
	After	20	9.2	44	20.2	154	70.6
6. Doping is not cheating since everyone does it.	Before	62	28.4	46	21.1	110	50.5
	After	19	8.7	36	16.5	163	74.8
7. Only the quality of performance should matter, not the way bodybuilders achieve it.	Before	72	33.0	47	21.6	99	45.4
	After	24	11.0	44	20.2	150	68.8
8. There is no difference between drugs and dietary supplements that are all used to enhance performance.	Before	29	13.3	31	14.2	158	72.5
	After	15	6.9	38	17.4	165	75.7

As per the Wilcoxon sign rank test, the average performance enhancement attitude levels before and after were significantly different in the study sample. The distribution of attitude scores were reduced from before to after the intervention with statistical significance in addition to median of agreement decreased for 7 out of 8 items ( Table 2).

**Table 2.** Performance enhancement attitude of bodybuilders before and after intervention.

Sl	Modified (for bodybuilding) Performance Enhancement Attitude Scale (PEAS) 8-item short form	Timing of survey to intervention	Number of responses	Median	p-value
1	Legalizing performance enhancements would be beneficial for sports or bodybuilding.	Before	218	Slightly Agree (4)	<0.001
		After	218	Strongly Disagree (1)	
2	Doping is necessary to be competitive.	Before	218	Slightly Disagree (3)	<0.001
		After	218	Disagree (2)	
3	The risks related to doping are exaggerated.	Before	218	Slightly Disagree (3)	<0.001
		After	218	Strongly Disagree (1)	
4	Bodybuilders should not feel guilty about breaking the rules and taking performance-enhancing drugs.	Before	218	Slightly Disagree (3)	<0.001
		After	218	Disagree (2)	
5	Doping is an unavoidable part of the competitive sport/bodybuilding.	Before	218	Slightly Agree (4)	<0.001
		After	218	Disagree (2)	
6	Doping is not cheating since everyone does it.	Before	218	Disagree (2)	<0.001
		After	218	Disagree (2)	
7	Only the quality of performance should matter, not the way bodybuilders achieve it.	Before	218	Slightly Disagree (3)	<0.001
		After	218	Disagree (2)	
8	There is no difference between drugs and dietary supplements that are all used to enhance performance.	Before	218	Disagree (2)	0.031
		After	218	Strongly Disagree (1)	

The median score for the pre-intervention survey was 24, and for the post-intervention survey was 14. The decrease in median score demonstrated a movement away from supporting use of performance enhancement substances and toward an anti-doping awareness.

## Discussion

The aim of this study was to assess any changes in the attitude of bodybuilders in the UAE about the use of supplements and doping, following the introduction of an antidoping educational flyer. The major finding was that there were significant changes in the attitudes of the study population to performance enhancement through supplement use and doping practices following pharmacy-led education with introduction of an antidoping flyer. Analysis of the responses to the eight-item PEAS survey revealed significantly different scores between pre- and post-intervention, suggesting less support for the use of performance enhancement substance among study participants. Using median, statistical tests proved the significance of the decrease in all eight survey items. Educational interventions and sensitization of sports people have shown similar results in the following studies.

It was found that doping susceptibility perceptions can be immediately reduced with educational interventions. The face-to-face intervention was observed to be more sustainable than online interventions among high-level athletes. ^20^ Nevertheless, a mobile application was found to be a practical method for disseminating anti-doping education. The application improved knowledge and decreased favorable doping attitudes among coaches. The mobile application had educational modules on nutritional supplements, substances, rules, leadership, and fair play. ^21^ Practical strength training advice provided in addition to anti-doping education to youth athletes was found beneficial in decreasing PEA. ^22^ PEA was found in many studies among gym users and athletes. Such studies proposed educational interventions to decrease PEA. ^7^^,^^8^^,^^23^

Sensitizing those who have potential for doping needs to start early, even in schools. Students who were professionally involved in sports perceived performance enhancement as more acceptable than other students. ^24^ Body image, nutritional supplement use, and weight change behaviors influence adolescents' PEA. ^25^ Media literacy interventions were effective to decrease doping behaviors of adolescent students. ^26^ It is accepted that attitudes and knowledge about doping are influenced by educational activity. ^27^^,^^28^ In addition, it is also possible that the studies itself had sensitized participants against PEA as assumed in our study.

## Conclusion

This study showed that the PEAS short-form survey, along with the introduction of an antidoping educational flyer provided by a sports pharmacy team, resulted in decreasing PEA among bodybuilding populations in the UAE. The decline of PEA was significant for all eight items of the PEAS. Bodybuilders, regardless of level of profession in the sport or level of competition, showed reduction in their attitude scores in the post-intervention survey. This study shows the potential for pharmacy-led educational interventions for bodybuilder athletes to influence favorable PEA, in support of the antidoping movement.

### Limitations

A limitation of the study was that, even though the UAE is a multicultural society, with English and Arabic being common languages, the survey was only conducted in English; therefore, the study population might not have fully represented the UAE population.

## Data Availability

OSF: Drug Use, https://doi.org/10.17605/OSF.IO/AUQX2. ^29^ This project contains the following underlying data:
• Bodybuilder survey.xlsx (This file contains raw data used for statistical analysis). Data are available under the terms of the Creative Commons Attribution 4.0 International license (CC-BY 4.0).

## References

[1] Loney T Aw TC Handysides DG : An analysis of the health status of the United Arab Emirates: the ‘Big 4’ public health issues. *Glob. Health Action.* 2013 Dec 1;6(1):20100. 10.3402/gha.v6i0.20100 23394856 PMC3566378

[2] Nawar R Ibrahim E Abusnana S : Understanding the Gaps in Obesity Management in the UAE: Perceptions, Barriers, and Attitudes. *Dubai Diabetes Endocrinol. J.* 2021;27(2):37–49. 10.1159/000514359

[3] Dalibalta S Majdalawieh A Yousef S : Objectively quantified physical activity and sedentary behaviour in a young UAE population. *BMJ Open Sport Exerc.* 2021 Jan 1;7(1):e000957. 10.1136/bmjsem-2020-000957 33489309 PMC7797257

[4] Yammine K : The prevalence of physical activity among the young population of UAE: a meta-analysis. *Perspect. Public Health.* 2017 Sep;137(5):275–280. 10.1177/1757913916675388 27810999

[5] Coquet R Roussel P Ohl F : Understanding the paths to appearance-and performance-enhancing drug use in bodybuilding. *Front. Psychol.* 2018;9:1431. 10.3389/fpsyg.2018.01431 30135676 PMC6092691

[6] Christiansen AV Vinther AS Liokaftos D : Outline of a typology of men’s use of anabolic androgenic steroids in fitness and strength training environments. *Drugs: Educ. Prev. Policy.* 2017 May 4;24(3):295–305. 10.1080/09687637.2016.1231173

[7] Sharif SI Mohammed A Mohammed I : Evaluation of knowledge, attitude and use of dietary supplements among people exercising in the gym in Sharjah-United Arab Emirates. *Phys. Med. Rehabil. Res.* 2018;3(5):1–5.31172033

[8] Abdulla NM Aziz F Blair I : Prevalence of, and factors associated with health supplement use in Dubai, United Arab Emirates: a population-based cross-sectional study. *BMC Complement. Altern. Med.* 2019 Dec;19(1):1–1. 31299957 10.1186/s12906-019-2593-6 PMC6624985

[9] Montuori P Loperto I Paolo C : Bodybuilding, dietary supplements and hormones use: behaviour and determinant analysis in young bodybuilders. *BMC Sports Sci. Med. Rehabil.* 2021 Dec;13(1):1–1. 10.1186/s13102-021-00378-x 34819149 PMC8613966

[10] Heller S Ulrich R Simon P : Refined analysis of a cross-sectional doping survey among recreational triathletes: Support for the nutritional supplement gateway hypothesis. *Front. Psychol.* 2020 Sep 23;11:561013. 10.3389/fpsyg.2020.561013 33071886 PMC7538671

[11] Backhouse SH Whitaker L Petróczi A : Gateway to doping? Supplement use in the context of preferred competitive situations, doping attitude, beliefs, and norms. *Scand. J. Med. Sci. Sports.* 2013 Mar;23(2):244–252. 10.1111/j.1600-0838.2011.01374.x 22092778

[12] AbdullGaffar B : Illicit injections in bodybuilders: a clinicopathological study of 11 cases in 9 patients with a spectrum of histological reaction patterns. *Int. J. Surg. Pathol.* 2014 Dec;22(8):688–694. 10.1177/1066896914553664 25311454

[13] Hearne E Wazaify M Van Hout MC : Anabolic-androgenic steroid use in the Eastern Mediterranean region: A scoping review of extant empirical literature. *Int. J. Ment. Health Addict.* 2021 Aug;19(4):1162–1189. 10.1007/s11469-019-00217-8

[14] Andreasson J Johansson T : Bodybuilding and fitness doping in transition. Historical transformations and contemporary challenges. *Soc. Sci.* 2019 Mar 4;8(3):80. 10.3390/socsci8030080

[15] Bates G Begley E Tod D : A systematic review investigating the behaviour change strategies in interventions to prevent misuse of anabolic steroids. *J. Health Psychol.* 2019 Sep;24(11):1595–1612. 10.1177/1359105317737607 29096544

[16] Petróczi A : Attitudes and doping: a structural equation analysis of the relationship between athletes' attitudes, sport orientation and doping behaviour. *Subst. Abuse Treat. Prev. Policy.* 2007 Dec;2(1):1–5. 10.1186/1747-597X-2-34 17996097 PMC2217289

[17] Bates G Van Hout MC Teck JT : Treatments for people who use anabolic androgenic steroids: a scoping review. *Harm Reduct. J.* 2019 Dec;16(1):1–5. 31888665 10.1186/s12954-019-0343-1 PMC6937954

[18] Petróczi A Aidman E : Measuring explicit attitude toward doping: Review of the psychometric properties of the Performance Enhancement Attitude Scale. *J. Sport Exerc. Psychol.* 2009 May 1;10(3):390–396. 10.1016/j.psychsport.2008.11.001

[19] Folkerts D Loh R Petróczi A : The Performance Enhancement Attitude Scale (PEAS) reached ‘adulthood’: Lessons and recommendations from a systematic review and meta-analysis. *J. Sport Exerc. Psychol.* 2021 Jun 8;56:101999. 10.1016/j.psychsport.2021.101999

[20] Nicholls AR Morley D Thompson MA : The effects of the iPlayClean education programme on doping attitudes and susceptibility to use banned substances among high-level adolescent athletes from the UK: A cluster-randomised controlled trial. *Int. J. Drug Policy.* 2020 Aug 1;82:102820. 10.1016/j.drugpo.2020.102820 32563179

[21] Nicholls AR Fairs LR Plata-Andrés M : Feasibility randomised controlled trial examining the effects of the Anti-Doping Values in Coach Education (ADVICE) mobile application on doping knowledge and attitudes towards doping among grassroots coaches. *BMJ Open Sport Exerc. Med.* 2020 Oct 1;6(1):e000800. 10.1136/bmjsem-2020-000800 33088583 PMC7547541

[22] Sagoe D Holden G Rise EN : Doping prevention through anti-doping education and practical strength training: The Hercules program. *Perform. Enhanc. Health.* 2016 Sep 1;5(1):24–30. 10.1016/j.peh.2016.01.001

[23] Al Nozha OM Elshatarat RA : Influence of knowledge and beliefs on consumption of performance-enhancing agents in north-western Saudi Arabia. *Ann. Saudi Med.* 2017 Jul;37(4):317–325. 10.5144/0256-4947.2017.317 28761032 PMC6150593

[24] Vargo EJ James RA Agyeman K : Perceptions of assisted cognitive and sport performance enhancement among university students in England. *Perform. Enhanc. Health.* 2014 Jun 1;3(2):66–77. 10.1016/j.peh.2015.02.001

[25] Yager Z O’Dea JA : Relationships between body image, nutritional supplement use, and attitudes towards doping in sport among adolescent boys: implications for prevention programs. *J. Int. Soc. Sports Nutr.* 2014 Mar 27;11(1):13. 10.1186/1550-2783-11-13 24670105 PMC3986904

[26] Lucidi F Mallia L Alivernini F : The effectiveness of a new school-based media literacy intervention on adolescents’ doping attitudes and supplements use. *Front. Psychol.* 2017 May 9;8:749. 10.3389/fpsyg.2017.00749 28536552 PMC5422551

[27] Deng Z Guo J Wang D : Effectiveness of the world anti-doping agency's e-learning programme for anti-doping education on knowledge of, explicit and implicit attitudes towards, and likelihood of doping among Chinese college athletes and non-athletes. *Subst. Abuse Treat. Prev. Policy.* 2022 Dec;17(1):1–3. 35473803 10.1186/s13011-022-00459-1 PMC9044811

[28] Wolff W Schindler S Brand R : The effect of implicitly incentivized faking on explicit and implicit measures of doping attitude: when athletes want to pretend an even more negative attitude to doping. *PLoS One.* 2015 Apr 22;10(4):e0118507. 10.1371/journal.pone.0118507 25902142 PMC4406708

[29] Thomas D : Drug Use.[Dataset].2023. 10.17605/OSF.IO/AUQX2

