# Supplementary material for: Impact of an educational flyer and sensitization on performance-enhancement attitudes of bodybuilders in United Arab Emirates
Source: F1000Res. 2024 Apr 16;12:208. Originally published 2023 Feb 23. [Version 2] doi: 10.12688/f1000research.130700.2 (PMC10964001; doi:10.12688/f1000research.130700.2)
Supplement: Peer review history

# Reviewer report: Reviewer response for version 1

**DOI:** 10.5256/f1000research.143473.r250949

**Published:** 2024-03-25

**Authors:** Nicholas Gibbs

**Affiliations:** 1 Department of Social Sciences, Northumbria University, Newcastle upon Tyne, England, UK

- I appreciated the background about physical culture and bodybuilding in the UAE and feel like the study was well-justified.
- The methodology of the work is sound, and the sample size is good enough for a small study like this, leading to credible results.
- However, I think that there is a blind spot in this paper about the willingness of IPED users to engage with medical services and pharmacists. Lots of literature has pointed to this and, although some improvements have been made with regard to building trust between the two communities, the question of who will actually see the flyer (those who actually engage with pharmacies) is pertinent. I’d like to see some understanding and caveat of that.
- How was the study advertised and how did recruitment actually take place? You say that you were ‘distributing a pre-intervention survey to registered study participants through email and WhatsApp’, but how did you get these participants’ details in the first instance?
- I’d like to see more on limitations of the study and a more tentative tone in your conclusion. Perhaps some acceptance that a qualitative aspect would bolster the data and a nod to the need for the flyer to be trailed for long-term effectiveness. By that I mean that, whilst your sample might have reported less inclination to use IPEDs immediately following the flyer, will this attitude be sustained, especially given the normalization of use in some communities?

Is the work clearly and accurately presented and does it cite the current literature?

Yes

If applicable, is the statistical analysis and its interpretation appropriate?

Not applicable

Are all the source data underlying the results available to ensure full reproducibility?

No source data required

Is the study design appropriate and is the work technically sound?

Yes

Are the conclusions drawn adequately supported by the results?

Yes

Are sufficient details of methods and analysis provided to allow replication by others?

Partly

Reviewer Expertise:

I am a qualitative social scientist with expertise in image and performance enhancing drug studies.

I confirm that I have read this submission and believe that I have an appropriate level of expertise to confirm that it is of an acceptable scientific standard, however I have significant reservations, as outlined above.

# Reviewer report: Reviewer response for version 1

**DOI:** 10.5256/f1000research.143473.r236837

**Published:** 2024-03-05

**Authors:** Valentin Panayotov

**Affiliations:** 1 Department of Weightlifting, Boxing, Fencing and Sports for all, National Sports Academy, Sofia, Bulgaria

In my opinion several inaccuracies should be addressed:

 1. A Bonferroni correction of the pre-post results should be performed.

 2. Kruskal Wallis test results are not presented and interpreted.

 3. " *When the average score is compared among the four groups by the median test, the median (average) of the overall prescore varied across the groups (p-value <0.001), but the average of the overall pre-score is almost the same between the groups (p-value 0.07). It implies that the post score changed significantly when compared with the average pre-score, but*

*the average attitude level became stable and is matched among different types of participants".* This paragraph is absolutely incomprehensible. It does not become clear whether the terms "among groups" and "across groups" mean "between groups" or "within groups". The results of the statistical procedures used should be explained in more detail and visualized by tables or/and graphs.

 4.In the results section the authors state that *" The distribution of attitude scores were reduced from before to after the intervention with statistical significance in addition to median of agreement decreased for 7 out of 8 items (Table 2)."* , but in the discussion and conclusion sections they assert that a decrease is measured in all eight items. This discrepancy should be addressed.

Is the work clearly and accurately presented and does it cite the current literature?

Yes

If applicable, is the statistical analysis and its interpretation appropriate?

Partly

Are all the source data underlying the results available to ensure full reproducibility?

Yes

Is the study design appropriate and is the work technically sound?

Yes

Are the conclusions drawn adequately supported by the results?

Partly

Are sufficient details of methods and analysis provided to allow replication by others?

Yes

Reviewer Expertise:

Sports science, dietetics, statistics, strength training

I confirm that I have read this submission and believe that I have an appropriate level of expertise to confirm that it is of an acceptable scientific standard, however I have significant reservations, as outlined above.

# Reviewer report: Reviewer response for version 1

**DOI:** 10.5256/f1000research.143473.r164451

**Published:** 2023-03-14

**Authors:** Narayana Goruntla

**Affiliations:** 1 Department of Clinical Pharmacy and Pharmacy Practice, Kampala International University, Ishaka, Western Region, Uganda

Overall, the study was novel, interesting, and well-designed, and drew reliable findings by application of suitable statistical methods. The study shows the significance of pharmacist-led interventions in lowering the doping property PEAS in bodybuilders. This study will serve as new evidence of pharmacist contribution to Antidoping. With a few modifications given below, the work can be processed for publication.

- In the background of the study, better to provide some evidence of generic names of drugs or chemicals that favors doping and causes health risks or complications.
- Though the PEAS short form is standard, validated, and internationally acceptable, the reliability of the tool in the study population can be given by providing Cronbach’s Alpha value. Because it was mentioned that the tool was modified according to the study need (applicable to bodybuilders and doping strategies).
- In the sample size determination, though the recommended sample size is acceptable, the margin of error needs to be less than or equal to 5%.
- In the results section, the total study population was divided into four groups, named non-sport employment bodybuilders, full-time bodybuilders, University students, and School students. It was mentioned that the median score was significantly different after intervention. There is a need to mention which group shows a high or low median score.

Is the work clearly and accurately presented and does it cite the current literature?

Yes

If applicable, is the statistical analysis and its interpretation appropriate?

Yes

Are all the source data underlying the results available to ensure full reproducibility?

Yes

Is the study design appropriate and is the work technically sound?

Yes

Are the conclusions drawn adequately supported by the results?

Yes

Are sufficient details of methods and analysis provided to allow replication by others?

Yes

Reviewer Expertise:

Pharmacoepidemiology, Pharmacoeconomics, Pharmacovigilance, Health Education, Epidemiology, Public health, Clinical Pharmacy, and  Pharmacy Practice.

I confirm that I have read this submission and believe that I have an appropriate level of expertise to confirm that it is of an acceptable scientific standard.
